# A New Porcine Reproductive and Respiratory Syndrome Virus with *N*-Linked Glycosylation Site Deletion in GP5 44th Amino Acid from JXA1, NADC30-Like, and JM Triparental Recombination

**DOI:** 10.1155/2023/4001055

**Published:** 2023-06-30

**Authors:** Xingdong Zhou, Sushu Bian, Enxi Kan, Lujia Zhou, Xiaohui Zhang, Min Xiao, Chang Lu, Ji Hua, Yuan Wu, Cheng Zhang, Yingshan Zhou, Wanyu Dong, Jing Du, Xiaodu Wang, Houhui Song

**Affiliations:** ^1^Key Laboratory of Applied Technology on Green-Eco-Healthy Animal Husbandry of Zhejiang Province, Zhejiang Provincial Engineering Laboratory for Animal Health Inspection & Internet Technology, Zhejiang International Science and Technology Cooperation Base for Veterinary Medicine and Health Management, China-Australia Joint Laboratory for Animal Health Big Data Analytics, College of Animal Science and Technology & College of Veterinary Medicine, Zhejiang A&F University, Hangzhou 311300, China; ^2^Jiangxi Zhengbang Academy of Agricultural Sciences, Nanchang 330029, China; ^3^Jinhua Polytechnic, Jinhua 321017, China; ^4^Hangzhou Zhengxing Animal Husbandry Co. Ltd., Hangzhou 311300, China

## Abstract

Porcine reproductive and respiratory syndrome virus (PRRSV) is a significant pathogen causing substantial financial losses in the global swine industry. The prevention of PRRSV is hampered due to frequent gene recombination among different strains of PRRSV. In this study, a new PRRSV strain, PRRSV-HQ-2020, was identified from nursery piglets in Yunnan Province, China, in 2020. The complete genome analysis revealed that PRRSV-HQ-2020 is highly similar to JXA1-like (lineage 8.7 PRRSV, isolated from China in 2008) in the 5′UTR, nsp1–9, and nsp11 coding regions. Additionally, it has a resemblance to JM (lineage 3 PRRSV, isolated from Taiwan, China, in 2010) in the nsp12-M coding region and NADC30 (lineage 1.8 PRRSV, isolated from North American in 2008) in the nsp10, N, and 3′UTR, suggesting a natural recombination event. Furthermore, recombination analyses showed three interlineage recombination events among lineages 8.7, 1.8, and 3. Notably, the GP5 protein of PRRSV-HQ-2020 exhibited a crucial mutation at position 44, leading to the deletion of a key glycosylation site. These findings provide direct evidence for the natural occurrence of recombination events among three lineages of PRRSV-2 in Chinese swine herds, leading to the emergence of unique genetic properties of PRRSV variants, and providing a theoretical basis for developing better PRRSV prevention strategies.

## 1. Introduction

Porcine reproductive and respiratory syndrome (PRRS) is a severe contagious ailment caused by porcine reproductive and respiratory syndrome virus (PRRSV) that has resulted in significant financial losses to the global swine industry over the past 3 decades [1, 2]. PRRSV isolates are categorized into two main genotypes based on their distinct genetic and antigenic variety: PRRSV-1 (European strains, EU) and PRRSV-2 (North American, NA), which have less than 60% genetic similarity [3]. According to phylogenetic analyses of PRRSV sequences, PRRSV-2 has been classified into nine monophyletic lineages [4].

PRRSV-2 strains have been prevalent in the Chinese swine industry for more than 2 decades [5, 6]. The most prevalent PRRSV-2 strains in China were mainly concentrated in the following four lineages, including lineages 1, 3, 5 (sublineage 5.1), and 8 (sublineage 8.7) [7]. As one of lineage 1 strain, NADC30-like PRRSV was first reported in Henan Province of China in 2013 [8, 9]. At present, sublineages 1.5 and 1.8 PRRSV have been gradually popular in China [10, 11]. Lineage 3 contains GM2-like PRRSV strains (QYYZ and GM2), which was first reported in 2010 and mostly endemic in Southeast China (Fujian, Guangdong, Guangxi, Taiwan) [1, 12, 13]. The sublineage 5.1 strain BJ-4 was isolated in 1997 and showed 99.6% of genome homology with VR2332 [14]. Sublineage 8.7 can be divided into five subgroups based on phylogenetic analysis of the ORF5 sequence: subgroup Ⅰ, subgroup ⅠⅠ, subgroup ⅠⅠⅠ, subgroup ⅠⅤ, and subgroup Ⅴ. Subgroup Ⅰ contains CH-1a, the first isolated PRRSV strain in China in 1996 and was recognized as an ancestor strain of classical PRRSV in China. Subgroup II was considered as an intermediate subgroup between classical PRRSV and HP-PRRSV. Subgroup Ⅲ–Ⅴ belongs to HP-PRRSV derivative strains [10, 15, 16].

Moreover, recombination has been identified as a strong driving force behind the evolution of PRRSV, in addition to amino acid deletions, insertions, and substitutions [17, 18]. The genetic recombination of PRRSV isolates between different lineages and sublineages in Chinese swine herds has been a topic of great interest and is a prevalent phenomenon in most countries [19]. As a result of recombination, clinical strains gradually exhibit increasing diversity and complexity, posing new challenges for PRRSV prevention and control. The coexistence of multiple subtypes and the possibility of virus recombination have gained widespread attention [18]. Since 2012, lineage 1 became the major parent of dominant recombination, and the recombinant strain with L1 as the backbone had strong viral replication ability [3]. Several severe reproductive and respiratory diseases have emerged in swine herds, with research suggesting that the pathogens responsible were new recombinant strains of PRRSV resulting from the combination of NADC30 strain with JXA1, HUN4-like strain, or vaccine strains [20–22]. Moreover, substantial changes in the genome were observed, including specific point mutations and deletions.

This study presents a new strain of PRRSV, which demonstrates mutations and recombination in its genome. The virus was isolated from a respiratory disease sample from the Heqing area. Through whole genome sequencing and software analysis, we found that the strain is a natural recombinant virus originating from three lineages (lineages 1.8, 3, and 8.7) of PRRSV-2 that circulate in China. Additionally, the recombinant strain contains a crucial amino acid mutation in GP5, which is a rarely reported finding. The strain can provide valuable scientific insight for the clinical prevention of PRRSV epidemics.

## 2. Materials and Methods

### 2.1. Sample Collection and Virus Isolation

During the routine epidemiological investigation of PRRSV, a lung sample was obtained from a farm that had been vaccinated with a live attenuated PRRSV in Yunnan Province, China, in 2020. The samples were positive for PRRSV and negative for porcine circovirus type 2, pseudorabies virus, classical swine fever virus, porcine parvovirus, Japanese encephalitis virus confirmed by reverse transcript PCR (RT-PCR) test. Subsequently, positive samples were collected, mixed with Dulbecco's modified Eagle's medium (DMEM), and grounded in a homogenizer. After centrifugation, the supernatants were passed through 0.22 *μ*m filters and inoculated into primary PAMs (obtained from specific pathogen-free piglets) and MARC-145 cells, respectively. The inoculated cells were cultured in an incubator at 37°C and 5% CO_2_ cytopathic effect (CPE) was observed. The virus was harvested through repeated freezing and thawing for further study and stored at −80°C. The PRRSV strain was confirmed by Western blot using a monoclonal antibody directed against the PRRSV N protein (which was kindly supplied by Professor Weihuan Fang of Zhejiang University) and actin (Proteintech, USA).

### 2.2. Viral Genome Extraction and RT-PCR

In accordance with the guidelines provided by the manufacturer, we employed TRIzol reagent (Ambion, USA) to extract viral RNA from the samples and used ReverTra Ace (Toyobo, Japan) to synthesize cDNA. To amplify viral genes, the full-length genome was divided into fragments (Table 1). The PCR reaction mixture of 50 *μ*l comprised of 25 *μ*l KOD, One PCR Master Mix-Blue (Toyobo, Japan), 4 *μ*l of corresponding primers (listed in Table 1), 2 *μ*l template, and 19 *μ*l distilled water. After predenaturation for 3 min at 98°C, 35 cycles of amplification (98°C for 10 s, 60–65°C for 5 s, and 68°C for 15–30 s) were performed, final extension at 68°C for 7 min.

The amplified PCR products were separated on a 1% agarose gel under ultraviolet light, and target fragments were extracted using Gel Extraction Kit (Huilin, Shanghai). The purified PCR products were then integrated into the pMDTM18-T vector (TAKARA, Japan), which was then transformed into *Escherichia coli* DH5*α* cells. The plasmids were extracted using Plasmid Extraction Mini Kit (Huilin, Shanghai). The Qingke Company (Hangzhou, China) completed the genomic sequencing, and the DNASTAR software Version 7.0 (DNASTAR Inc., Madison, USA) was used to assemble the sequence. Finally, the strain was named PRRSV-HQ-2020.

### 2.3. *Phylogenetic Analysis*, *Amino Acid Alignment*, *Glycosylated Analysis*, *and Recombinant Analysis*

To investigate the characteristics of the PRRSV-HQ-2020 strain, we performed multiple sequence alignments for nucleotides and amino acids using the clustal W method in MEGA 7.0 (AZ, USA) to determine sequence homology [23] (Table 2). The phylogenetic trees were constructed by MEGA 7.0 using neighbor-joining method with 1,000 bootstrap replicates for alignment, based on multiple sequences of PRRSV-2 available in GenBank (see *Supplementary 1* in the Supplementary Material for detail information) [24]. Interactive Tree Of Life (iTOL) (http://itol.embl.de/) was employed for phylogenetic tree annotation [25]. We predicted the glycosylation sites of the GP5 protein using the NetNGlyc 1.0 Server (https://services.healthtech.dtu.dk/service.php?NetNGlyc-1.0) [26]. To detect potential recombination events, we scanned the genomic sequences using a sliding window of 400 bp (20 bp step size) in Simplot (Version 3.5.1).

## 3. Results

### 3.1. Virus Isolation and Identification

**Figure 1:**
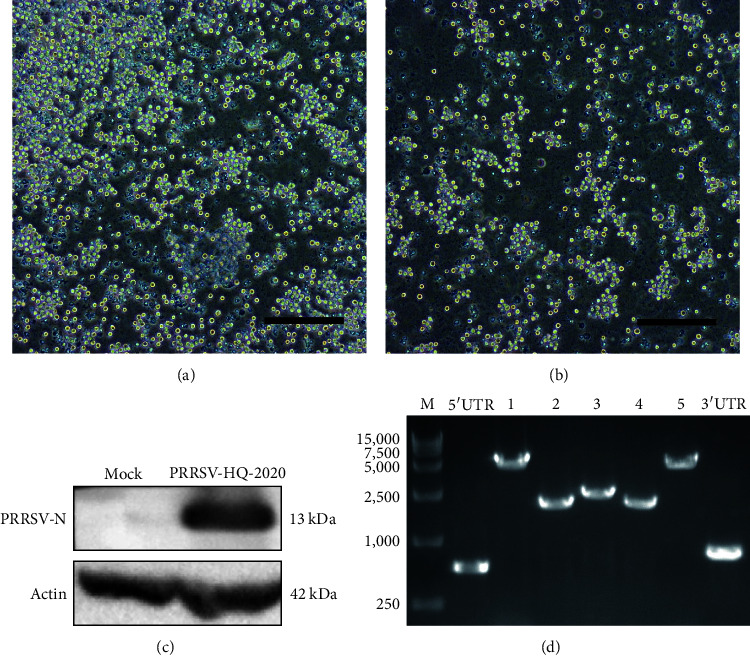
The results of PRRSV-HQ-2020 strain cytopathic. (a) Uninfected negative controls of PAMs. (b) PAMs inoculated with PRRSV-HQ-2020 after 48 hr. Scale bar = 50 *μ*m. (c) The presence of PRRSV-HQ-2020 was identified by Western blot using PRRSV N protein monoclonal antibody. (d) The RT-PCR results of PRRSV-HQ-2020 fragments amplified by full-length genomic sequencing primers (Table 1).

The samples were confirmed to be positive for PRRSV by RT-PCR test. Virus isolation was performed on the samples using porcine alveolar macrophages (PAMs) and MARC-145 cells. While no CPE was observed in MARC-145 cells, PAMs exhibited stable and typical CPE after 48 hr (as shown in Figures 1(a) and 1(b)). The purified strain obtained from these samples was named as PRRSV-HQ-2020 and was further confirmed through Western blot (Figure 1(c)). To obtain complete sequence of PRRSV-HQ-2020, fragments of full-length sequence were amplified by RT-PCR (Figure 1(d)).

### 3.2. Genomic Characteristic Analysis

The complete sequence of PRRSV-HQ-2020, genome has been uploaded in GenBank under accession no. ON142049. The complete genomic sequence spanned 15,321 base pairs, featuring a 189-nucleotide 5′ untranslated region and a 155-nucleotide 3′ untranslated region, with the poly(A) tail excluded. Upon comparative analysis, it was observed that PRRSV-HQ-2020 possessed 87.5% and 58.5% nucleotide sequence homology with the North American prototype VR-2332 and the European prototype Lelystad virus (LV), respectively. This indicates that PRRSV-HQ-2020 is a North American genotypic strain (PRRSV-2).

**Figure 2:**
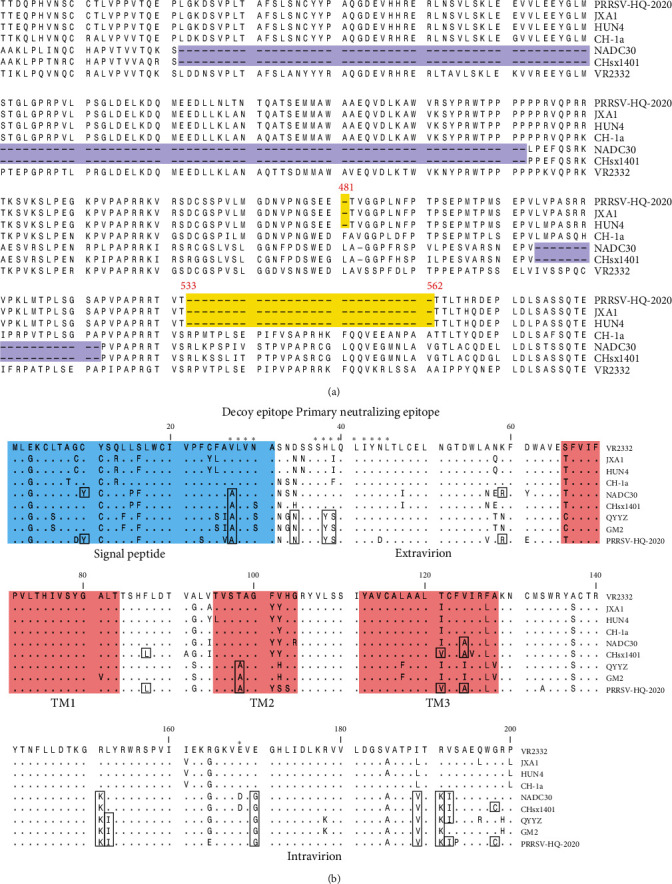
Alignment of the Nsp2 and GP5 protein amino acid sequence of PRRSV-HQ-2020 with representative PRRSV strains. (a) Alignment of partial Nsp2 protein aa sequences. Deletions in PRRSV-HQ-2020 and related virus strains are shaded in yellow, and deletions in NADC30-like strains are shaded in purple. (b) Alignment of amino acid mutations of GP5 protein. The signal peptide and transmembrane (TM) domains are demarcated and shaded. Asterisks indicate the regions of the decoy epitope and primary neutralizing epitope. The same amino acid sequences among PRRSV-HQ-2020, NADC30-like, and QYYZ-like are highlighted by black boxes.

The nonstructural protein NSP2 of PRRSV is known for its high variability, and the PRRSV-HQ-2020 strain exhibits varying patterns of insertions and deletions in this protein. NSP2 has been used as a reference genome for the research into the genetic variation and evolution of PRRSV. To further characterize the PRRSV-HQ-2020 strain, its NSP2 sequences were compared with those of reference PRRSV strains. Sequence alignment revealed that PRRSV-HQ-2020 contained two noncontinuous deletions in nsp2, a 1-amino acid deletion at position 281 and a 29-amino acid deletion at positions 533–562, in reference to the VR-2332 strain (Figure 2(a)). This deletion pattern of NSP2 in PRRSV-HQ-2020 was in line with that of most sublineage 8.7 strains (HP-PRRSV).

The genome ORF5, which encodes the structural protein GP5 of PRRSV, is known for its high variability. When compared with the reference strains, the PRRSV-HQ-2020 showed significant differences in both its extra-virion and intravirion regions. The amino acid analysis of PRRSV-HQ-2020 GP5, compared with other representative strains, revealed that it was more closely related to NADC30-like and QYYZ-like strains than to other strains (as shown in Figure 2(b)). The analysis also identified several unique amino acids such as 10Y, 59R, 87L, 121V, 124A, and 198 C, which were only identified in NADC30-like strains. In terms of the GP5 primary neutralizing epitope, there were three amino acid mutations (H38Y, L39S, and N44D), and in the decoy epitope, there was one mutation (V27A).

**Figure 3:**
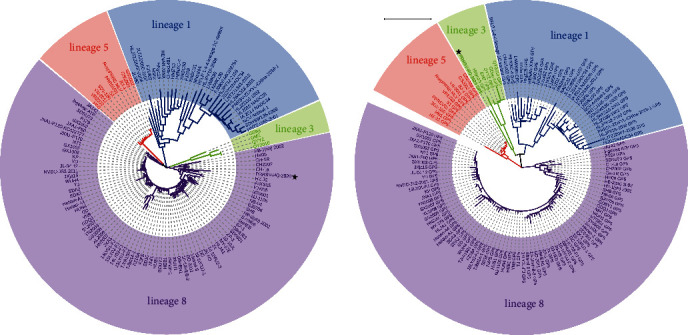
Complete genome-based (a) and GP5-based (b) phylogenetic tree of PRRSV-HQ-2020 and 123 reference PRRSV-2 strains. PRRSV-2 strains are divided into four lineages in both trees, which are shown in distinct colors. The recombinant virus PRRSV-HQ-2020 was clustered into a separate branch of lineage 8 in the complete genome-based phylogenetic tree and lineage 3 in GP5-based phylogenetic tree, marked with a black star. Each virus is presented by the virus name. Scale bar = 0.1 indicates the nucleotide substitution per site.

To further understand the genetic relationship between the PRRSV-HQ-2020 strain and other strains, a phylogenetic tree was constructed using both full-length genomic and GP5 nucleotide sequences of the PRRSV-HQ-2020 strain, along with 123 reference strains. The reference PRRSV-2 strains were found to be classified into four different lineages (lineages 8, 3, 5, and 1) in both the full-length genomic and GP5 phylogenetic trees, although there were differences in their distribution patterns. While the PRRSV-HQ-2020 strain was found to be located in different lineages (lineages 8 and 3) in different trees, it was located in a separate branch of the phylogenetic trees (as shown in Figure 3).

### 3.3. Recombination and Glycosylation Analysis

We conducted a comparative analysis of the nucleotide and amino acid sequences of various regions of the PRRSV-HQ-2020 genome with eight subgenotypes (I, III, V, and Ⅷ), identified by reference strains NADC30, WHU5, QYYZ, JM, VR2332, GM2, CH-1a, and JXA1, respectively (as shown in Table 3). Our findings revealed that the PRRSV-HQ-2020 isolate had high nucleotide identities (92.6%–100%) to JXA1 in 5′UTR, nsp1a-nsp9, and nsp11, but with lower nucleotide identities in nsp10, GP2-N, and 3′UTR (84.3%–90.1%). Additionally, the PRRSV-HQ-2020 strain shared the highest nucleotide identity with JM for nsp12-GP6. Based on these results, we conjecture that there may have been mosaic recombination events that occurred in the genomes of PRRSV-HQ-2020 isolate.

**Figure 4:**
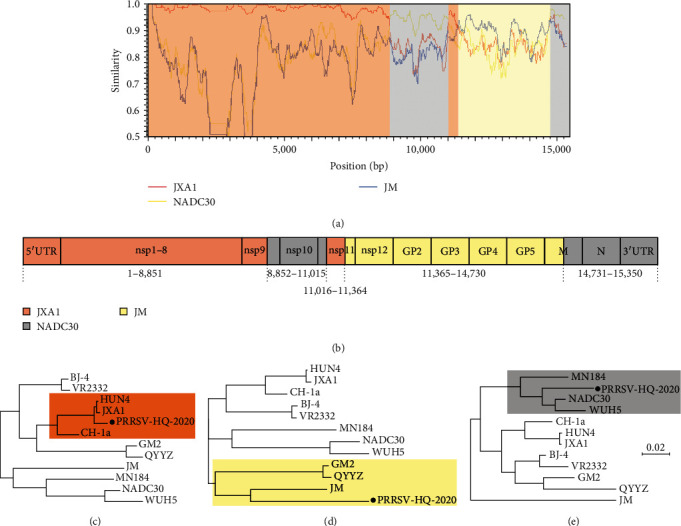
Genome recombination analysis of the PRRSV-HQ-2020 strain. (a) Recombination analysis of PRRSV-HQ-2020. (b) Schematic of the complete genome of PRRSV-HQ-2020. The phylogenetic trees were generated with the representative PRRSV genome. Tree (c) was inferred for the regions nt1–8851 and nt11085–11364. Tree (d) was inferred for the region nt11365 –14730. Tree (e) was inferred for the region nt8852–11015 and 14731–15350. Scale bar indicates the nucleotide substitution per site.

In order to gain a deeper understanding of the potential recombination events within the PRRSV-HQ-2020 genome, we performed similarity comparisons using a sliding window technique of 400 bp with a step size of 20 bp. Our analysis revealed that PRRSV-HQ-2020 was generally more closely related to the representative HP-PRRSV in China (JXA1) than to any other strains, as evidenced by the SimPlot graph (as shown in Figure 4(a)). However, three narrow regions were identified that exhibited disproportionately low levels of similarity between the two strains when compared to other regions. Interestingly, these three subregions of PRRSV-HQ-2020 displayed higher levels of similarity to lineage 3 PRRSV (JM) and lineage 1 PRRSV (NADC30) strains (Figure 4(a)). Based on these findings, we identified four potential recombination breakpoints in the PRRSV-HQ-2020 genome located in nsp9 (nt8851), nsp11 (nt11015, nt11364), and M (nt14730). These breakpoints divided the PRRSV-HQ-2020 genome into five distinct regions. The regions nt1–nt8852 and nt11016–nt11364 were closely related to the JXA1 strain, which is one of the earliest Chinese lineage 8.7 PRRSV strains (Figure 4(c)) The region nt11365–nt14730 was closely related to the JM strain, which is a Taiwan lineage 3 PRRSV strain in China (Figure 4(d)). Finally, the remaining part of the PRRSV-HQ-2020 genome was more closely related to NADC30, which is a North American lineage 1.8 PRRSV strain isolated in 2008 (Figure 4(e)). Our findings support the hypothesis that PRRSV-HQ-2020 is a natural recombinant of JXA1, NADC30, and JM, and that the virus may have gained genetic diversity through recombination with triparental PRRSV strains in China.

The glycosylation analysis of GP5 protein revealed that lineages 1 and 3 had three glycosylation sites (N34, N44, and N51, except for JM), while lineage 5 had four (N30, N33, N44, and N51). On the other hand, lineage 8 (except for PRRSV-HQ-2020) had five glycosylation sites (N30, N34, N35, N44, and N51) (as shown in Table 4). PRRSV-HQ-2020, as a lineage 8 strain, showed a distinctive glycosylation pattern with only three glycosylation sites (N33, N34, and N51) and a deletion of one site (N44) when compared to all other strains (except for parent strain JM). Furthermore, the origin N at position 44 was replaced by D when compared to the reference strain. A comprehensive image analysis is available in *Supplementary 2* in the Supplementary Material.

## 4. Discussion

PRRS is a highly significant disease of swine farming worldwide, caused by PRRSV, whose evolution has been rapid and persistent for a prolonged period. In China, PRRSV-2 has emerged as the predominant strain since 1995 and has caused multiple outbreaks, including HP-PRRSV, NADC30-like, QYYZ, and GM2, over the past 2 decades [4, 9, 27, 28]. The current prevalent PRRSV-2 isolates in China are classified into four lineages (lineages 1, 3, 5, and 8). Due to the low fidelity of viral RNA-dependent RNA polymerase (RdRP) and immune pressure selection, the simultaneous existence of different lineages of PRRSV-2 complicates the control of the disease in China and provides a suitable environment for PRRSV recombination, further challenging PRRSV epidemic prevention efforts [20].

In this study, we identified a novel PRRSV-2 strain, named PRRSV-HQ-2020, in Yunnan province. A genome-wide sequence analysis indicated that PRRSV-HQ-2020 belongs to North American genotypic strain (PRRSV-2). The glycosylation of viral envelope proteins is a well-known approach to viral immune evasion that aims to avoid, impede, and counterneutralizing antibodies [29]. Our research has discovered that the GP5 protein of the PRRSV-HQ-2020 strain lacks a vital glycosylation site (N44) commonly found in most PRRSV isolates, which may be related to the NGS specificity of its parent strain JM. Given the farm's vaccination, the new glycosylation pattern could play a potential role in the virus's biological characteristics and viral infection.

In the evolution of PRRSV, genetic recombination also plays an essential role in addition to mutations. The parental virus of PRRSV-HQ-2020 comprises JXAI, JM, and NADC30 isolates. In recent years, recombination events between HP-PRRSV and NADC30-like PRRSV have been frequently reported, resulting in recombinant viruses like SDhz1512 and SCcd17, which are recombination viruses among NADC30, HP-PRRSV, and classical isolates [8, 22, 30–32]. Recombination is a robust evolutionary force for PRRSV, and it contributes significantly to PRRSV replication and immunization resistance. Although several commercial PRRSV vaccines are currently available in the Chinese market, current modified live vaccines cannot provide complete cross-protection against heterologous PRRSV strains. The isolation of PRRSV-HQ-2020 enriches the PRRSV recombinant information database and serves as a reference for further research on the mechanism of recombinant strains and the biological characteristics of recombinant viruses. It also lays the groundwork for future research on vaccines with related mechanisms. Due to the specific changes of the PRRSV-HQ-2020 strain, further study on its pathogenicity and antigenicity is necessary. Our subsequent work will focus on the challenge experiments and immune protection experiments of the PRRSV-HQ-2020 strain.

## 5. Conclusions

To summarize, our research identified a new PRRSV-2 strain called PRRSV-HQ-2020. This strain is the result of recombination involving three distinct lineages and contains a glycosylation site deletion in GP5. Our findings demonstrate that recombination events naturally occur in Chinese swine herds and lead to the emergence of new PRRSV mutants with distinct genetic properties. Consequently, it may be necessary to update surveillance and vaccine strategies regularly to prevent the spread of PRRS.

## Figures and Tables

**Table 1:** Full-length genomic sequencing primers.

Primer	Sequence (5′-3′)	Length of amplicon (bp)	Primer location
5′UTR	GGAGTTTGGCGTAAACGCCCACCCCAGGG	571	1–571

1	CGTTTAGTGAACCGTATGACGTATAGGTGTTGGCTCTATGCCAC	4,821	1–4,821
	CCTCCCCCCTGAAGGCTTCGAAATTTGCGTGATCTTTAGTCCATTC		

2	GGTCAAAGTTTCCGCTATTCCATTTCGA	2,200	4,400–6,600
	ATCCAAGTCCTCGTCAGTGAGTCTCATG		

3	GCTCACACCATGGTCTGCGCAAGTTCTG	2,629	6,006–8,635
	TTAGTCACTGCACCGGACTGCGTGACCAAC		

4	AGGTGCCTTGAGGCTGATCTTGCATCTTGC	2,032	8,466–10,498
	GCGGAGAGAATCTACAACGCGCTTGTCTGTAG		

5	GACATGCCATCTTTGTGTATGACCCACATAGGCAAC	4,699	1,0321–15,020
	AATTTCGGCCGCATGGTTCTCGCCA		

3′UTR	CCCTGCCCACCACGTTGAAAGTGCCGCA	731	1,4619–15,350

**Table 2:** PRRSV strains used in this study.

Strain	Country	Year	Accession no.
NADC30	United States	2008	JN654459
WUH5	China (Wuhan)	2015	KU523366
VR2332	United States	1992	EF536003
BJ-4	China (Beijing)	1996	AF331831
QYYZ	China (Guangdong)	2011	JQ308798
JM	China (Taiwan)	2011	KP998410
CH-1a	China (Beijing)	1996	AY032626
JXA1	China (Jiangxi)	2006	EF112445
Lelystad virus	Netherlands	1991	M96262
HUN4	China (Hunan)	2006	EF635006
CHsx1401	China (Beijing)	2014	KP861625
GM2	China (Guangdong)	2011	Jn6624241

**Table 3:** Nucleotide (nt) and amino acid (aa) analysis of PRRSV-HQ-2020 and other representative PRRSV strains.

Genomic region	Sublineage 1				Sublineage 3				Sublineage 5				Sublineage 8			
	NADC30		WHU5		QYYZ		JM		VR2332		BJ-4		CH-1a		JXA1	
	nt	aa	nt	aa	nt	aa	nt	aa	nt	aa	nt	aa	nt	aa	nt	aa
5′UTR	93.1		91.5		95.8		–		94.8		48.7		97.9		**100.0**	
Nsp1a	89.3	96.7	86.5	92.8	92.6	96.1	87.4	94.5	90.9	95.0	91.1	95.6	96.1	96.7	**98.9**	99.4
Nsp1b	80.8	76.0	790	73.5	84.9	80.9	77.5	76.0	87.7	83.7	87.9	83.6	93.0	87.7	**98.4**	97.5
Nsp2	77.9	73.6	80.0	19.6	83.1	79.2	78.9	11.6	84.1	79.1	84.3	79.2	92.3	89.0	**98.7**	98.0
Nsp3	83.0	91.4	82.4	90.9	80.7	87.9	83.4	90.5	89.5	95.3	90.1	95.7	94.2	98.3	**98.6**	98.7
Nsp4	84.7	92.7	84.5	91.7	84.5	91.7	85.6	93.2	89.6	94.1	89.9	94.1	95.4	96.1	**99.5**	99.5
Nsp5	91.0	94.7	89.2	94.2	82.4	89.4	82.8	87.7	90.0	93.6	90.0	93.6	95.5	95.9	**99.0**	99.4
Nsp6	95.8	94.1	95.9	94.1	95.9	100.0	83.7	94.1	93.9	94.1	93.9	94.1	95.9	100.0	**98.0**	100.0
Nsp7	81.5	84.2	81.0	84.6	91.5	92.7	80.6	84.6	88.6	89.6	88.0	88.5	93.6	94.2	**96.8**	97.3
Nsp8	87.5	89.1	86.0	87.0	92.6	93.5	79.4	87.0	93.4	93.5	93.4	93.5	96.1	93.5	**96.3**	93.5
Nsp9	90.8	72.7	88.3	71.3	88.6	73.5	85.6	72.7	89.7	93.7	89.7	73.5	91.3	73.9	**92.6**	73.7
Nsp10	**93.6**	98.6	91.9	97.0	83.1	93.4	83.3	94.1	84.8	95.2	84.9	95.2	85.5	93.6	84.3	93.9
Nsp11	89.9	94.7	88.7	93.3	89.2	93.8	90.0	93.7	90.0	93.8	90.2	94.2	92.3	95.6	**93.9**	96.0
Nsp12	87.3	91.6	86.4	92.3	89.4	96.1	**93.5**	95.5	86.8	92.9	86.6	92.9	86.6	94.8	84.7	93.5
ORF2/GP2	84.2	82.6	83.8	82.2	86.4	84.9	**91.7**	89.5	87.2	84.5	87.4	85.3	87.6	84.9	86.1	84.1
ORF3/GP3	82.5	81.6	81.1	81.6	83.6	84.0	**88.0**	89.1	85.4	85.2	85.6	84.8	86.4	84.8	85.6	82.8
ORF4/GP4	86.6	90.0	85.3	87.8	87.5	90.0	**91.8**	95.0	88.5	90.0	88.5	90.0	89.0	92.8	88.1	91.7
ORF5/GP5	86.4	87.6	85.6	87.1	84.8	84.7	**86.9**	88.1	83.4	81.2	83.6	81.1	86.1	82.7	84.3	81.7
ORF6/M	90.5	94.3	89.4	93.8	89.7	93.2	**91.8**	94.3	90.9	93.8	90.7	93.1	89.4	92.6	89.9	93.2
ORF7/N	**95.7**	94.4	93.8	94.4	86.5	89.5	88.9	88.7	91.4	91.9	90.8	91.1	89.7	91.1	89.7	91.1
3′UTR	**98.0**		97.4		88.8		–		88.2		48.3		88.2		90.1	

Bold values signify the highest nucleotide identities of each genomic region.

**Table 4:** Comparation of potential *N*-glycosylation sites (NGSs) on positions 30–51 of GP5 protein.

Isolates	Lineage	*N*-Glycosylation sites						NGSs number
		N30	N33	N34	N35	N44	N51	
PRRSV-HQ-2020	8		+	+			+	3
JXA1	8	+		+	+	+	+	5
TJ	8	+		+	+	+	+	5
QYYZ	3			+		+	+	3
QY2010	3			+		+	+	3
JM^*∗*^	3							0
VR2332	5	+	+			+	+	4
BJ-4	5	+	+			+	+	4
NADC30	1			+		+	+	3
JL580	1			+		+	+	3

^*∗*^The NGSs of JM are at positions N52, N69, N130, N144, and N168 and none of which are at positions 30–51.

## Data Availability

All data generated or analyzed during this study are included in this published article and its supplementary information files.

## Supplementary Materials

Supplementary 1 Reference strains for phylogenetic tree construction.

Supplementary 2 The GP5 modeling of PRRSV-HQ-2020 strain. GP5 protein modeling was performed by AlfaFold 2.0 and shown as surface (cyan). The mutant amino acid residue was shown in yellow.
