# Supplementary material for: A New Porcine Reproductive and Respiratory Syndrome Virus with N-Linked Glycosylation Site Deletion in GP5 44th Amino Acid from JXA1, NADC30-Like, and JM Triparental Recombination
Source: Transbound Emerg Dis. 2023 Jun 30;2023:4001055. doi: 10.1155/2023/4001055 (PMC12016986; doi:10.1155/2023/4001055)
Supplement: Supplementary 1 — Reference strains for phylogenetic tree construction. [file 4001055.f1.pdf]

Table S1 Reference strains for phylogenetic trees construction.

No.	Virus strain	Origin	Accession No.	Year
1	ATCC VR2332	USA	U87392	1992
2	RespPRRS MLV	USA	AF066183	1994
3	SDSU73	USA	JN654458	1996
4	JA142	USA	AY424271	1997
5	lngelvac ATP	USA	DQ988080	1999
6	MN184A	USA	DQ176019	2001
7	P129	USA	AF494042	2002
8	NADC30	USA	JN654459	2008
9	NADC31	USA	JN660150	2008
10	IA/2014/NADC34	USA	MF326985	2014
11	IA/2014/NADC35	USA	MF326986	2015
12	RFLP 1-4-4 lineage 1C variant	USA	MW887655	2020
13	CH-1a	China	AY032626	1996
14	BJ-4	China	AF331831	1996
15	S1	China	DQ459471	1999
16	HB-1(sh)/2002	China	AY150312	2001
17	HB-2(sh)/2002	China	AY262352	2001
18	GS2003	China	EU880442	2003
19	HN1	China	AY457635	2003
20	NB-04	China	FJ536165	2004
21	SHB	China	EU864232	2005
22	JXA1	China	EF112445	2006
23	HUN4	China	EF635006	2006
24	TJ	China	EU860248	2006
25	HEB1	China	EF112447	2006
26	HUB1	China	EF075945	2006
27	BJ0706	China	GQ351601	2007
28	GD	China	EU825724	2007
29	WUH1	China	EU187484	2007
30	Henan-1	China	EU200962	2007
31	NM1	China	EU860249	2007
32	GDQJ	China	GQ374441	2007
33	NT0801	China	HQ315836	2008
34	CH-1R	China	EU807840	2008
35	JXA1-p80	China	FJ548853	2008
36	HN2007	China	EU880437	2008
37	SX2007	China	EU880434	2008
38	XL2008	China	EU880436	2008
39	YN2008	China	EU880435	2008
40	GS2008	China	EU880431	2008
41	PRRSV01	China	FJ175687	2008
42	KP	China	GU232735	2008
43	YN9	China	GU232738	2008
44	JN-HS	China	HM016158	2008

45	GDBY1	China	GQ374442	2008
46	JXA1-P120	China	KC422727	2009
47	JXA1-P170	China	JQ804986	2009
48	ZP-1	China	HM016159	2009
49	SD1-100	China	GQ914997	2009
50	GS2002	China	EU880441	2009
51	CH2002	China	EU880438	2009
52	YD	China	JF748717	2009
53	SX-1	China	GQ857656	2009
54	SY0909	China	HQ315837	2009
55	09HEB	China	JF268679	2009
56	09HEN1	China	JF268684	2009
57	09HUB1	China	JF268682	2009
58	SD0901	China	JN256115	2009
59	DC	China	JF748718	2010
60	GX1003	China	JX912249	2010
61	QY2010	China	JQ743666	2010
62	10-10HEB-3	China	JQ663553	2010
63	Shanxi-6	China	KJ855518	2010
64	GX1001	China	JQ955657	2010
65	10-10FUJ-2	China	JQ663547	2010
66	10-10FUJ-1	China	JQ663546	2010
67	JX	China	JX317649	2010
68	SDA3	China	JX878380	2011
69	SDA2	China	JX878379	2011
70	WUH4	China	JQ326271	2011
71	YN-2011	China	JX857698	2011
72	GM2	China	JN662424	2011
73	QYYZ	China	JQ308798	2011
74	GD-2011	China	KC527830	2011
75	NJ-1106	China	JX880029	2011
76	NVDC-JS2-2011	China	JQ715698	2011
77	HH08	China	JX679179	2011
78	HZ-31	China	KC445138	2012
79	GX1002	China	JQ955658	2012
80	SD16	China	JX087437	2012
81	10-10JL	China	JQ663554	2012
82	JL-04/12	China	JX177644	2012
83	FJFS	China	KP998476	2012
84	NT1	China	KP179402	2012
85	MY-376	China	KJ609517	2013
86	Henan-A4	China	KJ534539	2013
87	HeNan-A1	China	KJ002451	2013
88	HENAN-XINX	China	KF611905	2013
89	HLJA1	China	KT351739	2013
90	FJZ03	China	KP860909	2013
91	FJW05	China	KP860911	2013
92	FJ1402	China	KX169191	2014

93	CHsx1401	China	KP861625	2014
94	14LY01-FJ	China	KP780881	2014
95	14LY02-FJ	China	KP780882	2014
96	TJbd14-1	China	KP742986	2014
97	GDsg	China	KX621003	2015
98	FJXS15	China	KX758250	2015
99	TJnh1501	China	KX510269	2015
100	HNjZ15	China	KT945017	2015
101	HNyc15	China	KT945018	2015
102	JL580	China	KR706343	2015
103	HENXC-4	China	KU950371	2015
104	JXja15	China	KR149645	2015
105	15LY01-FJ	China	KU215416	2015
106	15LY02-FJ	China	KU215417	2015
107	FZ16A	China	KY761966	2016
108	HNhx	China	KX766379	2016
109	LNWK130	China	MG913987	2018
110	FJ0908	China	MK202794	2018
111	PRRSV-ZDXYL-CHina-2018-1	China	MK453049	2018
112	HLJZD30-1902	China	MN648055	2018
113	LNWK96	China	MG860516	2018
114	NCV-Anheal-1	China	MH370474	2018
115	HLJWK318-2001	China	OL516357	2020
116	HNTZJ165-2001	China	OL516358	2020
117	LNTZJ1341-2012	China	OL516360	2020
118	HLJPY18-2009	China	OL516347	2020
119	SDHSW135-2009	China	OL516361	2020
120	JS2021NADC34	China	MZ820388	2021
121	JLTZJ2050-2107	China	OL516359	2021
122	HLJTZJ2007-2106	China	OL516354	2021
123	HLJPY32-2109	China	OL516348	2021
