# Supplementary material for: A New Porcine Reproductive and Respiratory Syndrome Virus with N-Linked Glycosylation Site Deletion in GP5 44th Amino Acid from JXA1, NADC30-Like, and JM Triparental Recombination
Source: Transbound Emerg Dis. 2023 Jun 30;2023:4001055. doi: 10.1155/2023/4001055 (PMC12016986; doi:10.1155/2023/4001055)
Supplement: Supplementary 2 — The GP5 modeling of PRRSV-HQ-2020 strain. GP5 protein modeling was performed by AlfaFold 2.0 and shown as surface (cyan). The mutant amino acid residue was shown in yellow. [file 4001055.f2.pdf]

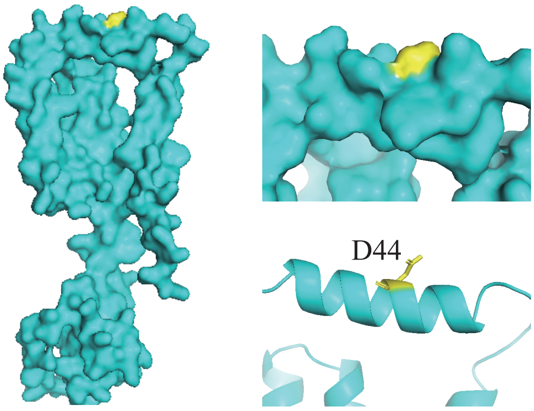

Figure S1 The GP5 modeling of PRRSV-HQ-2020 strain. GP5 protein modeling was performed by AlfaFold 2.0 and shown as surface (cyan). The mutant amino acid residue was shown in yellow.
